# Genetic architecture of protein expression and its regulation in the mouse brain

**DOI:** 10.1186/s12864-021-08168-y

**Published:** 2021-12-04

**Authors:** Alyssa Erickson, Suiping Zhou, Jie Luo, Ling Li, Xin Huang, Zachary Even, He Huang, Hai-Ming Xu, Junmin Peng, Lu Lu, Xusheng Wang

**Affiliations:** 1 Department of Biology, University of North Dakota, Grand Forks, ND 58202 USA; 2 Center for Proteomics and Metabolomics, St. Jude Children’s Research Hospital, Memphis, TN 38163 USA; 3 Central Laboratory, Zhejiang Academy of Agricultural Sciences, Hangzhou, 310021 China; 4 Institute of Bioinformatics, College of Agriculture and Biotechnology, Zhejiang University, Hangzhou, 310058 China; 5 Department of Genetics, Genomics and Informatics, University of Tennessee Health Science Center, Memphis, TN 38163 USA

**Keywords:** Proteome, Mouse, Brain, Protein expression, Allele-specific expression, Protein regulation, Mass spectrometry

## Abstract

**Background:**

Natural variation in protein expression is common in all organisms and contributes to phenotypic differences among individuals. While variation in gene expression at the transcript level has been extensively investigated, the genetic mechanisms underlying variation in protein expression have lagged considerably behind. Here we investigate genetic architecture of protein expression by profiling a deep mouse brain proteome of two inbred strains, C57BL/6 J (B6) and DBA/2 J (D2), and their reciprocal F1 hybrids using two-dimensional liquid chromatography coupled with tandem mass spectrometry (LC/LC-MS/MS) technology.

**Results:**

By comparing protein expression levels in the four mouse strains, we observed 329 statistically significant differentially expressed proteins between the two parental strains and characterized the genetic basis of protein expression. We further applied a proteogenomic approach to detect variant peptides and define protein allele-specific expression (pASE), identifying 33 variant peptides with *cis*-effects and 17 variant peptides showing *trans*-effects. Comparison of regulation at transcript and protein levels show a significant divergence.

**Conclusions:**

The results provide a comprehensive analysis of genetic architecture of protein expression and the contribution of *cis*- and *trans*-acting regulatory differences to protein expression.

**Supplementary Information:**

The online version contains supplementary material available at 10.1186/s12864-021-08168-y.

## Background

One of the fundamental goals of biological research is to understand the genetic basis of phenotypic variation. The phenotypic variation is substantially contributed by the regulation of gene expression at both transcriptional and protein levels [1]. Previous studies revealed that the regulation of gene expression is ubiquitous in humans and other organisms and is controlled by the interplay between genetic and environmental factors [2, 3]. The regulation of gene expression at the cell-type and single-cell level has also recently been investigated owing to advances in single-cell transcriptomics [4, 5]. While proteins are more relevant to phenotypic variation than transcripts, the regulation of protein expression has lagged behind considerably.

Recently, liquid chromatography coupled with tandem mass spectrometry (LC-MS/MS) technology has become a powerful platform for profiling deep proteomes, enabling us to investigate the regulation of protein expression. Genome-wide analysis of mRNA and protein expression in mice revealed a discrepancy between their regulations using quantitative trait loci (QTLs) mapping [3, 6]. In addition, allele-specific expression (ASE) is also used to further dissect the regulation into *cis-* and *trans-* components. Although ASE at the transcript level has been extensively explored and pervasive allelic imbalance across different tissues was identified [7, 8], only one study to date examined protein allele-specific expression (pASE) in yeast using stable isotope labeling by amino acids in cell culture (SILAC) technology [9].

Recombinant inbred (RI) strains are a useful resource for identifying genetic variation in phenotypic traits [10]. The BXD RI panel, derived from C57BL/6 J (B6) and DBA/2 J (D2), exhibits high and uniform levels of genetic and phenotypic variation [11]. Genetic regulation of gene expression and ASE at the transcript level have been studied in multiple tissues from the BXD RI panel. For example, over 50% of transcripts showing ASE in the liver were detected in the two reciprocal F1 hybrids (B6D2F1 and D2B6F1) [12, 13]. However, the transcript level is often not an accurate indicator of protein abundance [14, 15]. More importantly, genetic inheritance of protein expression and protein ASE (pASE) are not well defined.

**Fig. 1:**
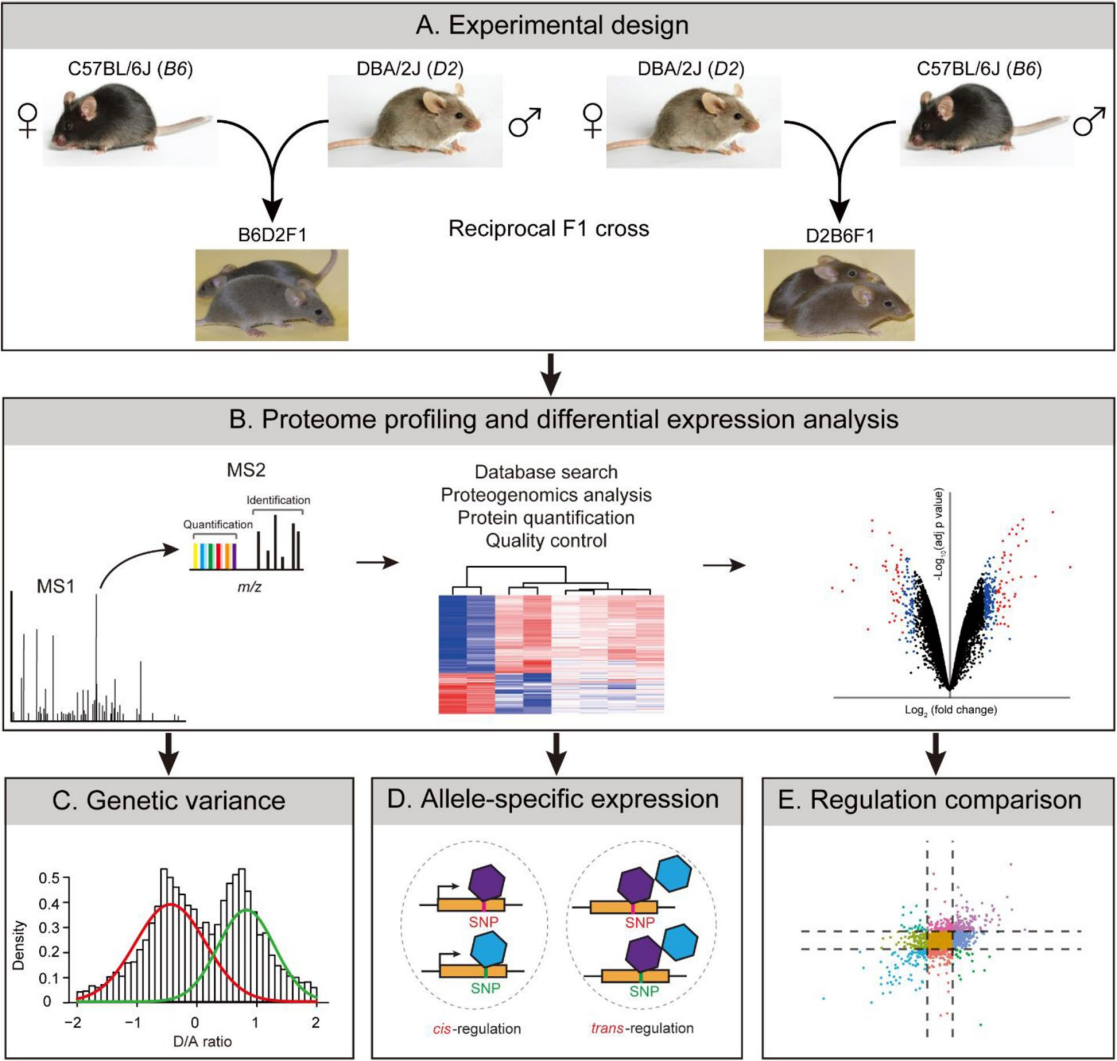
Schematic diagram of the experimental design and data analysis of this study. **A** Experimental scheme. Four strains, including B6, D2, and the two reciprocal F1s (B6D2F1 and D2B6F1), were used. **B** Mouse brain proteome was profiled by TMT-based proteomics, followed by data quality control and differential expression analysis. **C** Genetic variance analysis. **D** Allele-specific expression was defined by the proteogenomics approach. **E** Regulations at the transcript and protein levels were compared

To characterize the genetic regulation of protein expression, we first perform deep proteome profiling of brain tissue from B6 and D2 mouse strains, as well as their two reciprocal F1 hybrids using 11-plex tandem mass tag (TMT)-based LC/LC-MS/MS (Fig. 1A). We then detect differentially expressed proteins between the two parental strains (Fig. 1B) and characterize genetic basis of protein expression (Fig. 1C). We finally define *cis-* and *trans-*regulation of protein expression using the proteogenomics approach (Fig. 1D) and examine the difference in the regulation at transcript and protein levels (Fig. 1E).

## Results

### Comprehensive and quantitative proteome profiling of the mouse brain

**Fig. 2:**
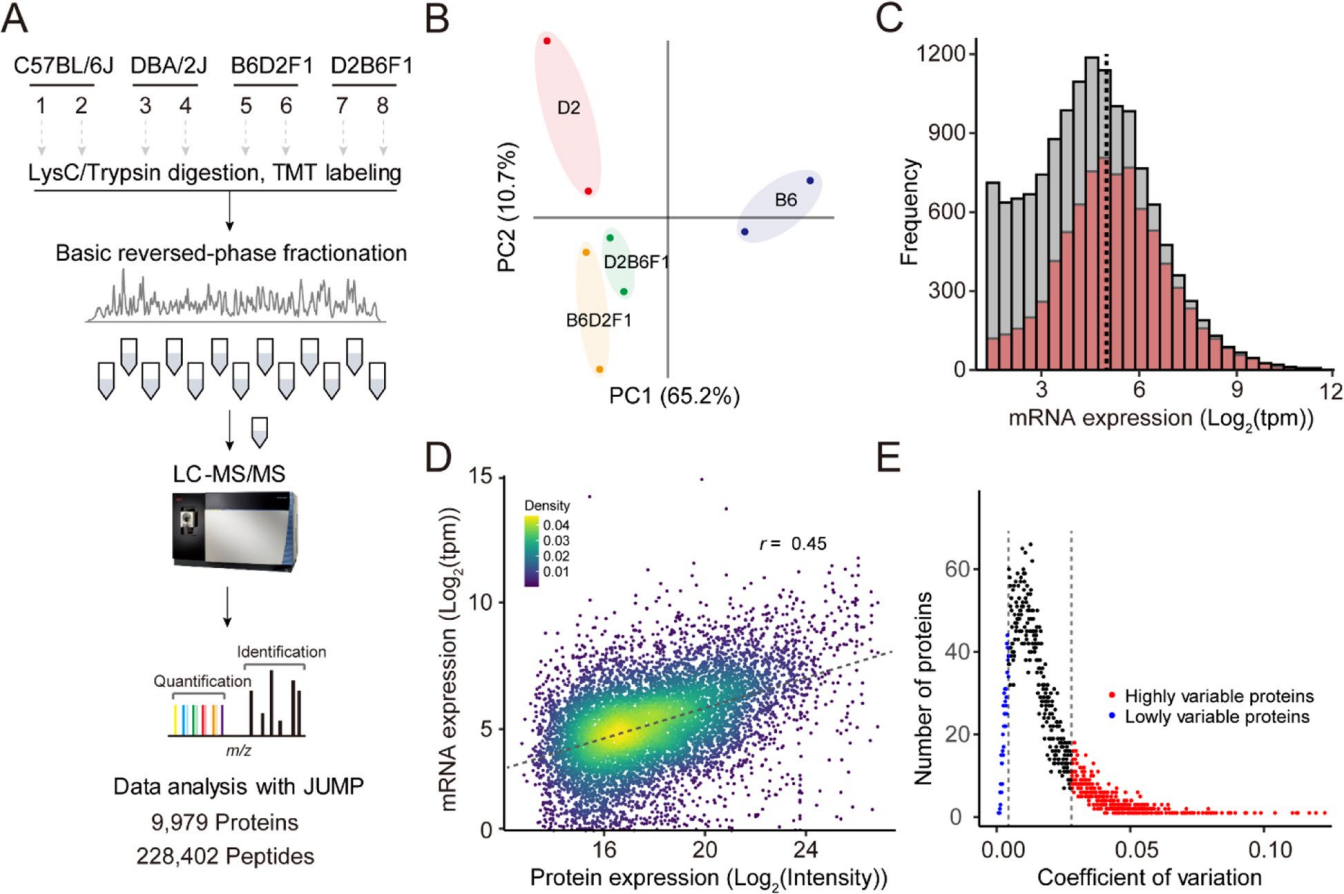
Proteome-wide profiling of mouse brain tissue. **A** 10-plex TMT-based global proteome analysis workflow. A total of 10 samples were analyzed by LC/LC-MS/MS. All proteomic data were analyzed using JUMP software. More than 228,000 distinct peptides, corresponding to 9979 proteins, were identified and quantified. **B** Principal-component analysis of all quantified proteins. **C** Histogram showing the coverage of proteomic data compared to RNAseq data from B6 and D2 mice. The open bar represents the distribution of protein coding genes detected by RNAseq, whereas the red bar indicates the distribution of protein coding genes from proteomic data. **D** Scatter plot showing a comparison of absolute expression between proteins and protein-coding transcripts. **E** Distribution of coefficient of variation (CV) for all proteins

To characterize genetic architecture of protein expression, we generated a deep brain proteome of four mouse strains, including B6 and D2, and their two reciprocal F1 hybrids (i.e., B6D2F1 and D2B6F1) (Fig. 2A). By using 11-plex TMT-based LC/LC-MS/MS with extensive fractionation, we identified a total of 273,063 peptide-spectrum matches (PSMs) and 87,892 peptides, corresponding to 9979 proteins (9688 genes) at protein FDR < 1% (Fig. 2A; Additional file 1: Table S1). Principal component analysis shows that two replicates of four mouse strains grouped well (Fig. 2B). Pearson correlation analysis also shows a high correlation (*r*^*2*^ = ~ 0.99) between the two replicates (Additional file 2: Fig. S1). The agreement of biological replicates indicates a high quality of the proteomic data.

We next ask to what extent mRNA expression can be detected by our proteomic data and whether there are differences between mRNAs across four mouse strains reflected at the level of proteins. To this end, we compared our proteomic data with transcriptome data from the same mouse strains generated by RNA sequencing [7, 8] with respect to protein coverage and absolute abundance (i.e., concentration). The proteins identified in this study cover most (80.5%) of highly abundant genes (i.e., log_2_(tpm) > 5), indicating deep coverage of the expressed proteome (Fig. 2C). Consistent with previous reports [14, 15], comparison of absolute abundance showed a modest correlation between mRNA and proteins (correlation coefficient *r*^2^ = 0.459; *p* value < 2.2 × 10^− 16^) (Fig. 2D). The discrepancy between proteins and mRNAs could be ascribed to protein translation rates and post-translational modifications as well as biases of RNA-sequencing and mass spectrometry technologies.

Genetic variation can lead to the difference in expression level of the same protein across different mouse strains. To determine which proteins are influenced by the genetic variation, we calculated the coefficient of variation (CV) across all four strains for each protein. We found that a subset of proteins (1347/9979) showed high variation in protein expression (Fig. 2E), defined as two standard deviations above the average of the CV. Gene Ontology (GO) enrichment analysis showed that these variable proteins were enriched in chromatin modification and protein secretion.

### Genetic difference in protein expression

**Fig. 3:**
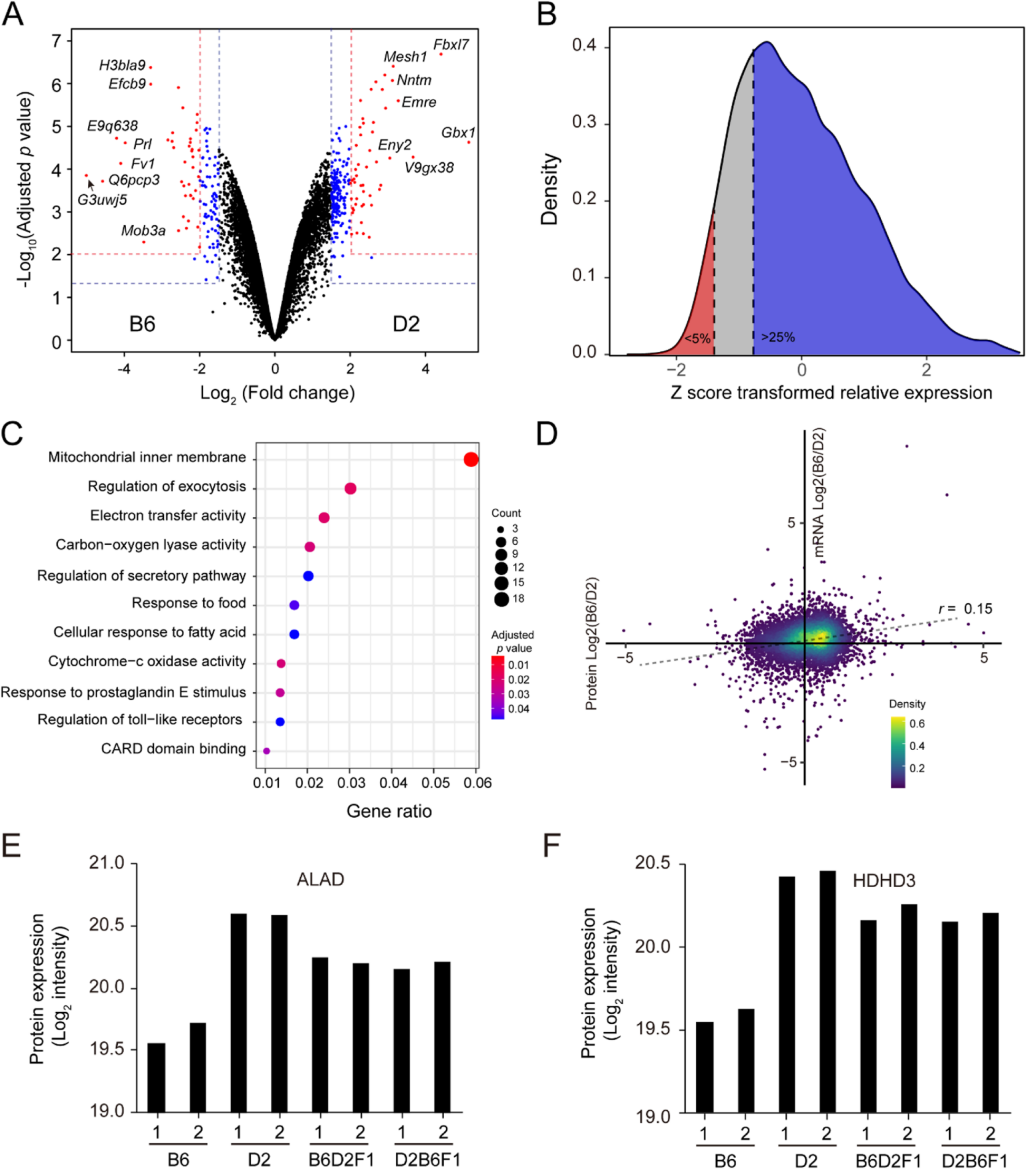
Analysis of differentially expressed (DE) proteins between B6 and D2. **A** The volcano plot of the differentially expressed proteins. Log_2_ fold change was plotted against the −log10 adjusted *p*-value with two criteria: (1) 4-fold change and 1% FDR; (2) 2-fold change and 5% FDR. **B** Distribution of z-score transformed relative expression between B6 and D2 for all 9979 proteins. An extreme form of differential expression was used to define single-parent expression. **C** Enrichment analysis of DE proteins. **D** Scatter plot showing a comparison of relative expression between proteins and protein-coding transcripts. **E**-**F** Expression levels of ALAD and HDHD3 between B6, D2, and the two F1s

We next sought to identify differentially expressed proteins (DEPs) between B6 and D2 strains, as they are highly polymorphic in genotypes and phenotypes. In our data, we identified 329 DEPs at the FDR of 0.05 and log2 fold change (log_2_FC) cut-off of 1.5 (Fig. 3A; Additional file 2: Fig. S2), including 113 and 216 proteins with higher expression in B6 and D2, respectively (Additional file 1: Table S2). A large proportion (71.4%) of proteins show a modest level of expression alteration (log_2_FC between 1.5 and 2). Among the 322 out of 329 DE proteins on autosomes, we identified 25 proteins with single parent expression (SPE), defined as an extreme form of differential expression in which either B6 or D2 shows a high expression abundance (log_2_ expression level z-score > 25th percentile) while the other is silent (log_2_ expression level z-score < 5th percentile) (Fig. 3B). Gene ontology enrichment analysis of all 329 DEPs displayed the highest enrichment for the cellular component of mitochondrial inner membrane, suggesting a difference in mitochondrial function between the two strains (Fig. 3C, Additional file 1: Table S3). Enrichment analysis performed on the 113 and 216 DEPs with higher expression in B6 and D2, respectively, revealed that DEPs with higher relative expression in D2 were significantly enriched for terms related to mitochondrial function, electron transfer activity, and cytochrome-c oxidase activity (Additional file 2: Fig. S3). In contrast, DEPs with higher relative expression in B6 were significantly enriched for GO terms related to prostaglandin response; however, this enrichment was driven by the three proteins, AKAP8, GNAS, and P2RY4.

We also define a correlation of the relative expression (B6 vs. D2) between mRNA and protein levels. We observed a fairly low correlation of relative expression ratio in mRNA compared to protein (Fig. 3D), indicating potential buffering at the protein level caused by genetic variation. Despite their low correlation, we confirmed consistent changes at both transcript and protein levels, such as ALAD and HDHD3 (Fig. 3E, F), for which, in our previous study, we found that ALAD and HDHD3 span with a copy number variation (CNV) and are associated with high variation in mRNA expression in multiple brain regions between B6 and D2 strains [11].

**Fig. 4:**
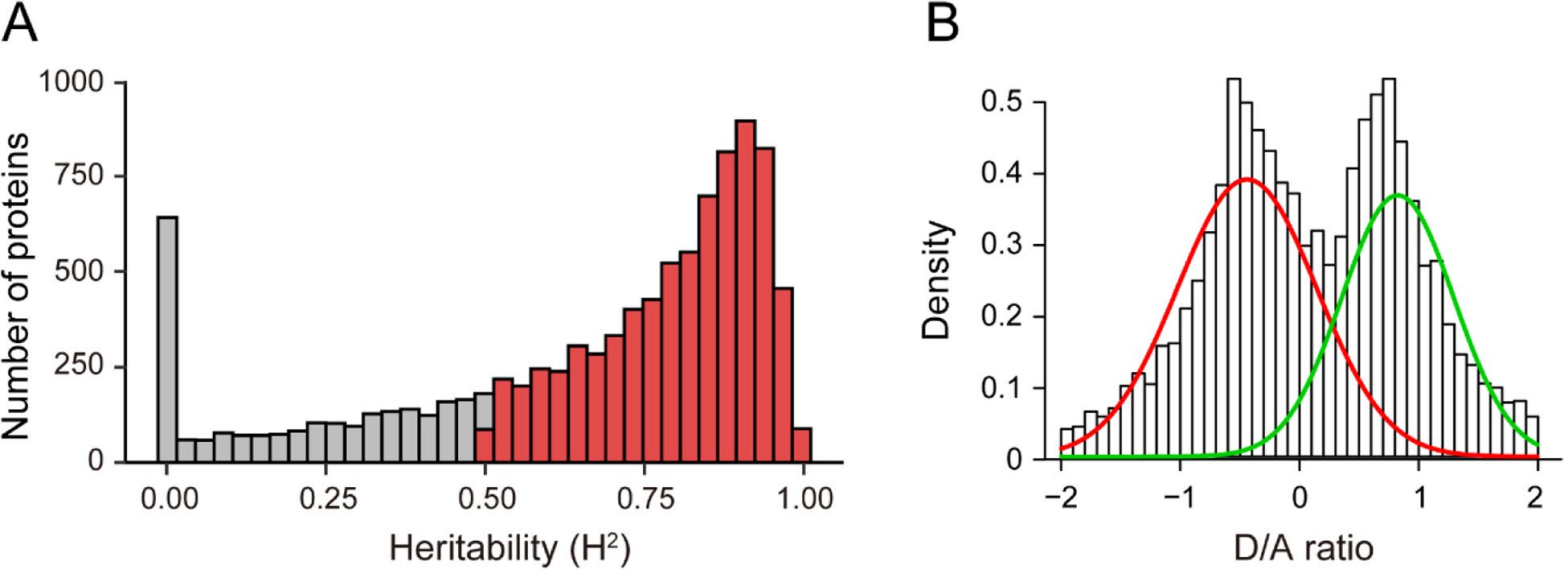
Patterns of genetic inheritance in protein expression. **A** Distribution of broad-sense heritability (H^2^) of protein expression. **B** Distribution of dominance to additivity (D/A) ratio. Dominance is the difference between the observed F1 transcript abundance (in this case averaged over the two reciprocal F1 genotypes) and the midpoint of the two parents. Additivity is the absolute value of the difference between the parental means relative protein abundance

The differences in protein expression across strains can be further partitioned into heritable and non-heritable variation. To calculate the heritability, we considered the genetic relatedness for both additive and dominant variances. The proportion of heritable variation (genetic) contributing to the total observed variation is known as the broad-sense heritability (H^2^). The median heritability estimate among all expressed proteins is 77% (Additional file 1: Table S4), which is higher than that of transcripts in BXD strains [16]. We found that 7594 (76.1% of the total) proteins showed heritability > 50% (Fig. 4A). We evaluated the inheritance patterns of protein expression using the distribution of D/A (i.e., dominance/additivity), revealing that the dominant expression pattern is more common than the additive pattern (Fig. 4B).

### Identification of protein allele-specific expression in *cis*- and *trans*-regulations

The highly variable genome sequences between B6 and D2 strains provide an opportunity to investigate allele-specific expression. While several studies have investigated ASE in mice using transcriptomics data, there is no research for pASE. Recently, the proteogenomics approach that integrates genomic and proteomic data has been proven to be a valuable method in detecting variant peptides [17–19]. We performed the proteogenomics analysis to detect variant peptides using JUMPg, a proteogenomics pipeline we recently developed.

**Fig. 5:**
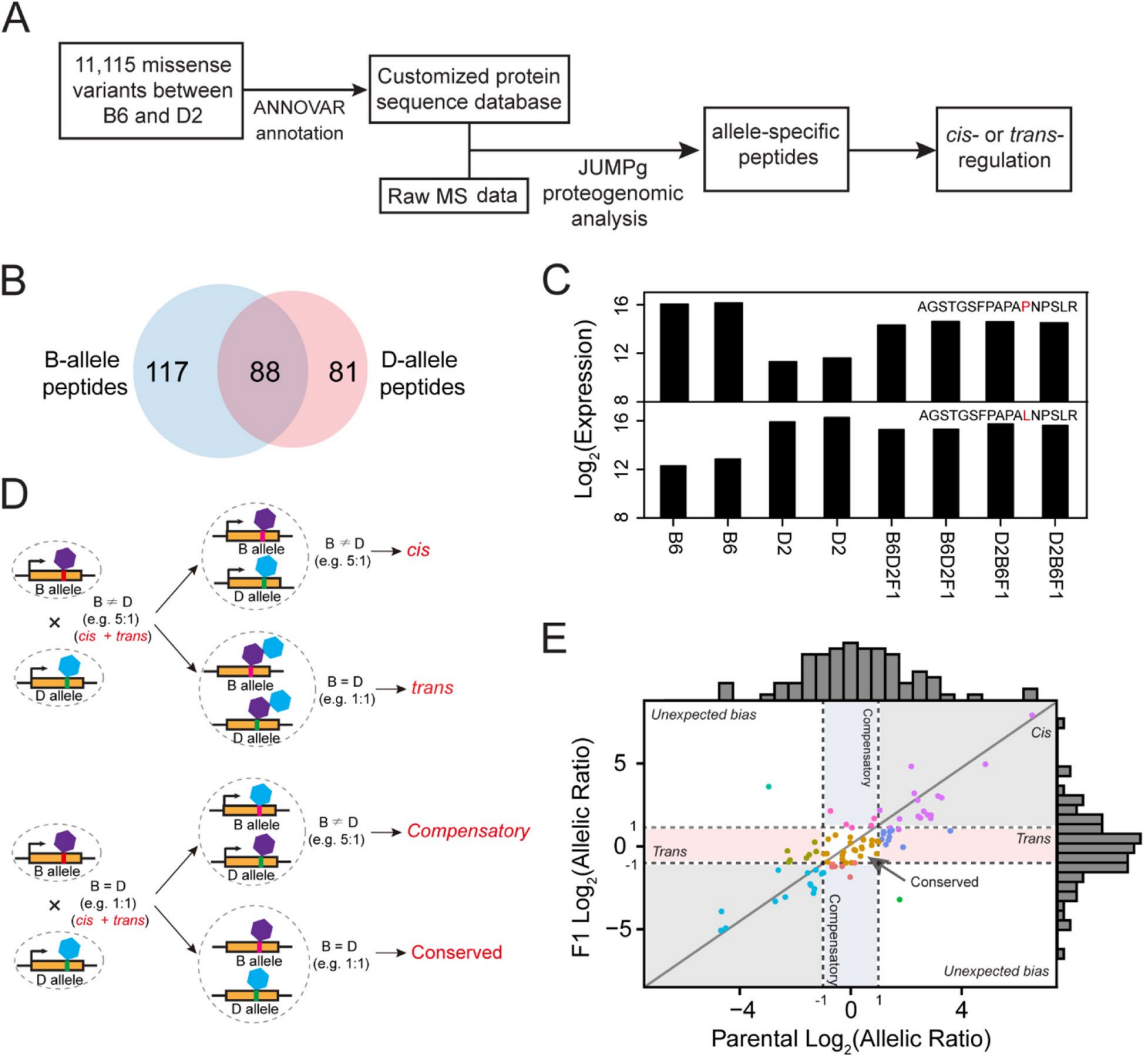
Protein allele-specific expression. **A** Workflow of allele-specific expression detection using the proteogenomic approach. **B** Venn diagram showing the overlap number of B-type and D-type variant peptides. **C** Two examples showing expression pattern of B-type and D-type variant peptides. **D** Conceptual diagram of *cis*-, *trans*-, compensatory, and conserved regulation. **E** Scatterplot of protein allelic ratios in parental and F1 strains showing different regulations: *cis*-, *trans*-, compensatory, and conserved

Using 11,115 missense variants detected in our previous D2 sequencing project, we identified a total of 286 variant peptides, including 169 and 205 D-allele and B-allele peptides, respectively, at the peptide FDR of 1% (Fig. 5A; Additional file 1: Table S5). By comparing B-allele and D-allele peptides, we found a total of 88 pairs of variant peptides (Fig. 5B, Additional file 1: Table S6). Two examples of B-allele peptide and D-allele peptide are shown in Fig. 5C. The B-allele peptide can only be detected in the B6 strain and both F1 hybrids, whereas the D allele peptide can be detected in the D2 strain and both F1 hybrids. Even though the signal of the two allelic peptides cannot be directly compared due to different chemical properties that alter ionization efficiency, the ratio of the two alleles in parents and F1 hybrids can be calculated, allowing us to determine pASE. By comparing the allelic ratio in parents and F1 hybrids, the regulation of protein expression is classified into five different categories: *cis*-, *trans*-, compensatory, conserved, and unexpected bias (Fig. 5D). The ratio of the two parental alleles is contributed by a combination of *cis*- and *trans*-regulatory effects. If the allelic ratio in the F1 is similar to the parental proportions, the differential expression in the parents is likely due to variation in *cis*-acting elements because the common *trans*-effect is present in the F1 hybrids. In contrast, if the change in the allelic ratio is only observed in parental strains but not in F1s (log_2_ ratio < 1), it is likely to be caused by *trans*-acting factors. If there is no change in parental strains, but with significant changes in F1 hybrids, the *cis*- and *trans*- effects in the parental are compensatory. We define conserved regulation as there are no changes in both parental strains and F1 hybrids.

Among those 88 pair variant peptides with both B- and D-types (Fig. 5E), we found 33 displaying *cis*-regulation, followed by 25 with conserved regulation. In addition, 17 displayed *trans*-regulation and 5 compensatory regulation. In addition, two proteins showed unexpected bias, which could be due to peptide false identification or quantitative measurement error in the shot-gun proteomics.

### Comparison of regulations at transcript and protein expression levels

**Fig. 6:**
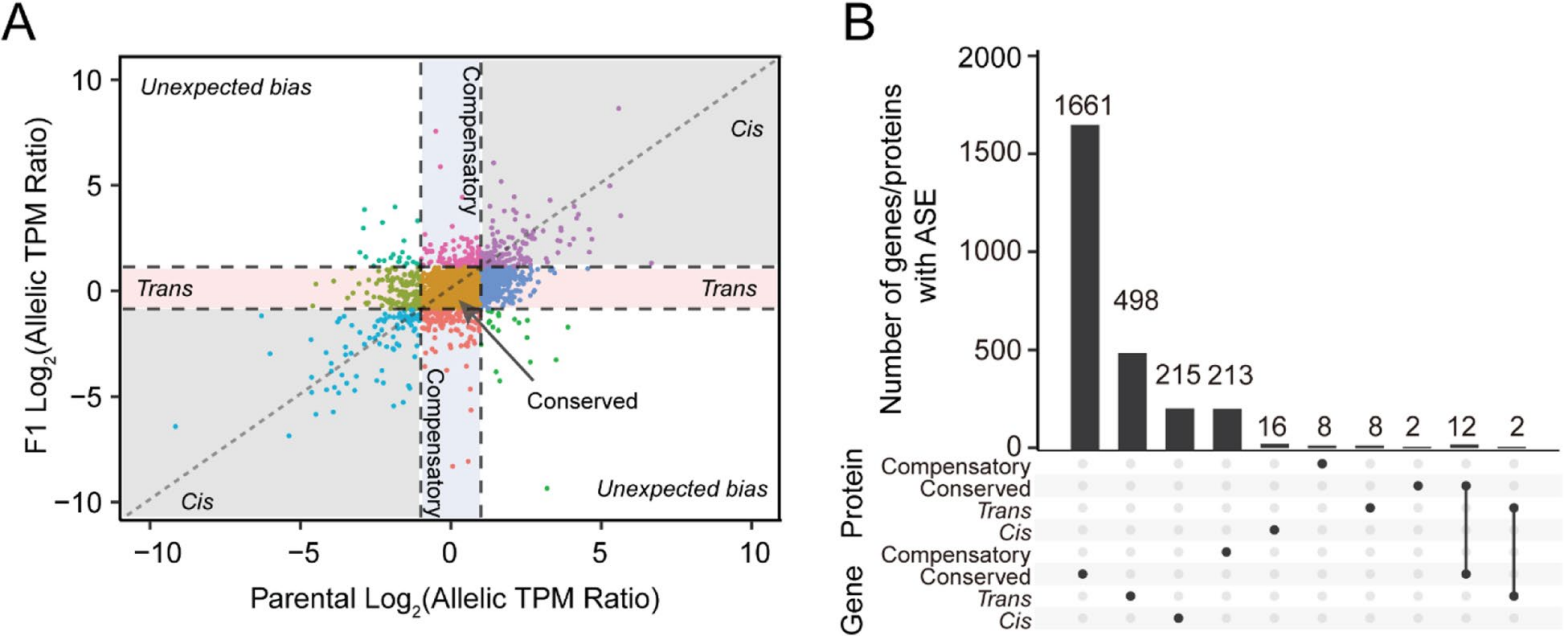
Comparison of allele-specific expression at transcript and protein levels. **A** Scatterplot showing regulations at the transcript level. **B** UpSet plot showing the number of different regulations between transcript and protein levels

We next sought to compare regulation at transcript and protein expression levels. To identify ASE at the transcript level, we analyzed the transcriptome of the hippocampus of matched samples (i.e., B6, D2, D2B6F1, and B6D2F1). By mapping RNA-seq data to both B6 reference and D2 customized genomes, we identified 2630 protein-coding transcripts with ASE expression (Fig. 6A), including 215 *cis*-, 500 *trans*-, 213 compensatory, 1666 conserved, and 36 unexpected bias regulation. By comparing regulation between transcripts and proteins, we found that there is a significant overlap between transcripts and proteins showing ASE (Fisher’s exact test *p* = 2.2 × 10^− 9^). The conserved regulation showed the highest overlapped in both levels. However, only two genes/proteins were found to show ASE in *trans*-regulations (Fig. 6B).

## Discussion

In this study, we profiled the mouse brain proteome of B6 and D2 strains, as well as their two reciprocal F1 hybrids, allowing us to investigate the regulation of protein expression. With this deep proteomic data, we identified 329 DEPs between B6 and D2 strains and 25 proteins with SPE. We further estimated broad-sense heritability for all 9979 proteins identified. We finally defined the allele-specific expression and *cis*- and *trans*-regulation of variant peptides detected by the proteogenomics approach. The deep proteomic data provides a unique opportunity to investigate the genetic regulation of protein expression in the mouse brain.

Our experimental design of including two inbred mice and their F1 hybrids enabled us to analyze the heterosis of protein expression. As expected, we found the two F1s are highly correlated (*r* = 0.99; *p* value = 1 × 10^− 5^) (Additional file 2: Fig. S4A). By comparing expression levels between B6D2F1 and D2B6F1, we observed 47 proteins with significant change in expression (*p* value < 0.01 and Fold change > 0.3), including 31 and 16 proteins showed up- and down-regulated, respectively. The relative expression of the two hybrids allows us to identify proteins with imprinted expression. We found 6 out of 47 proteins are found to be potentially associated with imprinting status including NLRP5, AFX15, TMSB10, GAP43, BUD23, and HMGA1 (Additional file 2: Fig. S4B). For example, NLRP5 (MATER) is a single-copy gene expressed exclusively in oocytes and has previously been described as a maternal-effect gene in mice [20]. However, surprisingly, we observed only 25 proteins exhibiting single-parent expression (SPE) at the protein level. In contrast, previous studies found 347 genes showing SPE at the transcript level in the mouse brain [21]. One possible reason for this discrepancy is that divergent translation regulation buffers mis-expression of mRNA abundance [22]. In addition, TMT ion suppression in the shot-gun proteomics could alleviate the difference in protein expression.

In the GO analysis of DEPs between B6 and D2, the most significantly enriched GO term was regulation of exocytosis (*p* = 5.7 × 10^− 6^). The main contributing genes are derived from regulation of synaptic vesicle (STXBP2, DOC2b, and SCAMP5), intracellular signal transduction (STAM and GLRX), axon elongation (ADORA2B and TSG101), and key regulators of presynaptic function (SYT7). This observation is consistent with previous proteomics studies in the mouse brain that have shown reduced expression of exocytosis proteins in D2 [23]. Cellular response to fatty acid is also found to be enriched in DEPs (*p* = 9.6 × 10^− 5^), which is in agreement with constitutive differences in regional brain fatty acid composition between B6 and D2 [24].

Compared with gene ASE analysis of transcriptomic data, protein ASE has not been well studied and only one study to date examined it in yeast using SILAC technology [9]. Instead of using the SILAC approach, we used a TMT-based approach to quantify the expression of variant peptides. There are several advantages of the TMT method over the SILAC when applied to the pASE study: (1) it is capable of including a larger number of samples that can be compared, which can increase protein coverage and reduce the batch effect. For example, we used 10 samples in one batch of a TMT experiment, whereas it requires at least five batches for a SILAC experiment; (2) since isobaric labeling methods (i.e., TMT, iTRAQ) are a chemical labeling approach, they can be applied to human samples and are less expensive to use in small mammals.

While the proteogenomics approach has been widely used to detect variant peptides [17–19], we extended its application to defining ASE for variant peptides detected in quantitative proteomic data. Compared with the fact that ASE can be defined by read counts at the transcript level, one of the major challenges in defining protein *cis*- and *trans*-regulation is that the expression level of variant peptides cannot be directly compared because they have different amino acid compositions altering their mass and ionization efficiency, which significantly complicates the accurate analysis of pASE. In this study, we propose to define the regulation using relative ratios of expression level between two types of variant peptides in F1 hybrids and their expression in their two parental strains.

One of the limitations of this study is the number of ASE events detected at the protein/peptide levels due to the constraints of the current shot-gun proteomics technology. While we generated, to the best of our knowledge, the deepest proteome coverage (~ 10,000 unique proteins) in mice, we only detected 374 variant peptides and 88 shared B-type and D-type variant peptides out of 11,115 missense genomic variants detected from the whole genome sequencing. The number is also substantially fewer than the number of variants (2630) detected from RNA-seq based transcriptomic data. With further advances in mass spectrometry technology, it will be possible to improve the ability to detect more variant peptides and ultimately define genome-wide pASE events.

Another limitation of this study is the small sample size for each biological sample. In a TMT-based quantitative proteomics experiment, it is of paramount importance to have adequate statistical power to minimize the false positive identification rate. This statistical power is influenced by sample size and effect size. Although a small sample size (*n* = 2) was used in this study, we believe that we can detect a similar number of differentially expressed proteins (DEPs) compared to other studies (~ 3000–5000 proteins) as we generated extreme deep protein coverage (~ 9000) using two-dimensional liquid chromatography coupled to mass spectrometry (i.e., 40 fractions), which was demonstrated by a simulation study (Additional file 2: Fig. S5). The deep proteome allows us to detect a reasonable number of differentially expressed proteins with a small sample size.

## Conclusion

In summary, our study provides a framework for investigating allele-specific expression. Allele-specific expression could be caused by epigenetic regulation, such as methylation. Further investigations are needed to understand the mechanisms underlying allele-specific expression by methylome profiling. Further investigation may provide important insights into the pathways that protein allele-specific expression contributes to phenotypic and disease variation. With the development of cell-type proteomics technology [25], genetic regulation of protein expression and pASE will be eventually defined at the cell-type or even single-cell level.

## Methods

### Animals

The following mouse strains were used in this study: C57BL/6 J (B6) and DBA/2 J (D2), and two reciprocal F1 hybrids (i.e. B6D2F1 and D2B6F1). The B6D2F1 hybrid is created by mating a B6 female to a D2 male mouse, whereas the D2B6F1 is created by mating a D2 female to a B6 male mouse. Both male and female mice of each strain were used as biological replicates in this study (*n* = 2). B6 and D2 mice were purchased from JAX (stock number 000664 and 000671, respectively). The two reciprocal F1 hybrids (B6D2F1 and D2B6F1), were bred at the University of Tennessee Health Science Center (UTHSC). Animals were housed and maintained on a 12: 12 light/dark cycle, with ad libitum access to food and water. Mice at 12-week-old were sacrificed, and whole brain tissue samples (Left and right olfactory bulbs were removed) were dissected rapidly, frozen in liquid nitrogen, and stored at − 80 °C for the subsequent proteome profiling. The euthanasia was carried out by cervical dislocation. Criteria for euthanasia were based on an assessment by our veterinary staff following AAALAC guidelines.

### Protein extraction and quantification

The frozen samples were weighed and homogenized in the lysis buffer (50 mM HEPES, pH 8.5, 8 M urea, and 0.5% sodium deoxycholate, 100 μl buffer per 10 mg tissue) with 1x PhosSTOP phosphatase inhibitor cocktail (Sigma-Aldrich). Protein concentration was measured by the BCA assay (Thermo Fisher) and then confirmed by Coomassie-stained short SDS gels.

### Protein digestion and tandem-mass-tag (TMT) labeling

Quantified protein samples (∼1 mg in the lysis buffer with 8 M urea) for each TMT channel were proteolyzed with Lys-C (Wako, 1:100 w/w) at 21 °C for 2 h, diluted by 4-fold to reduce urea to 2 M for the addition of trypsin (Promega, 1:50 w/w) to continue the digestion at 21 °C overnight. The digestion was terminated by the addition of 1% trifluoroacetic acid. After centrifugation, the supernatant was desalted with the Sep-Pak C18 cartridge (Waters), and then dried by Speedvac. Each sample was resuspended in 50 mM HEPES (pH 8.5) for TMT labeling, and then mixed equally, followed by desalting for the subsequent fractionation. For whole proteome analysis alone, 0.1 mg protein per sample was used.

### Extensive two-dimensional liquid chromatography-tandem mass spectrometry (LC/LC-MS/MS)

The TMT labeled samples were fractionated by offline basic pH reverse phase LC, yielding 40 fractions. Each fraction was analyzed by the acidic pH reverse phase LC-MS/MS [26]. In the acidic pH LC-MS/MS analysis, each fraction was run sequentially on a column (75 μm × 20 cm for the whole proteome, 50 μm × ∼30 cm for phosphoproteome, 1.9 μm C18 resin, 65 °C to reduce backpressure) interfaced with a Q Exactive HF Orbitrap or Fusion MS (Thermo Fisher). Peptides were eluted by a 2–3 h gradient (buffer A: 0.2% formic acid, 5% DMSO; buffer B: buffer A plus 65% acetonitrile). MS settings included the MS1 scan (410–1600 m/z, 60,000 or 120,000 resolution, 1 × 10^6^ AGC and 50 ms maximal ion time) and 20 data-dependent MS2 scans (fixed first mass of 120 m/z, 60,000 resolution, 1 × 10^5^ AGC, 100–150 ms maximal ion time, HCD, 35–38% normalized collision energy, ∼1.0 m/z isolation window).

### Identification of proteins by database search with JUMP software

We used JUMP search engine [27] to search MS/MS raw data against a composite target/decoy database to evaluate FDR [28]. All original target protein sequences were reversed to generate a decoy database that was concatenated to the target database. FDR in the target database was estimated by the number of decoy matches (nd) and the number of target matches (nt), according to the equation (FDR = nd/nt), assuming mismatches in the target database were the same as in the decoy database. The target database was downloaded from the UniProt mouse database (59,423 entries), and decoy database was generated by reversing targeted protein sequences. Major parameters included precursor and product ion mass tolerance (±15 ppm), full trypticity, static mass shift for the TMT tags (+ 229.16293) and carbamidomethyl modification of 57.02146 on cysteine, dynamic mass shift for Met oxidation (+ 15.99491), maximal missed cleavage (*n* = 2), and maximal modification sites (*n* = 3). Putative PSMs were filtered by mass accuracy and then grouped by precursor ion charge state and filtered by JUMP-based matching scores (Jscore and ΔJn) to reduce FDR below 1% for proteins during the whole proteome analysis. If one peptide could be generated from multiple homologous proteins, based on the rule of parsimony, the peptide was assigned to the canonical protein form in the manually curated Swiss-Prot database. If no canonical form was defined, the peptide was assigned to the protein with the highest PSM number.

### TMT-based peptide/protein quantification by JUMP software suite

Protein expression was quantified using the following steps with JUMP software suite: (i) TMT reporter ion intensities were extracted for each PSM; (ii) the raw intensities were corrected based on isotopic distribution of each labeling reagent; (iii) PSMs with very low intensities (e.g. minimum intensity of 1000 and median intensity of 5000) were excluded from the subsequent analysis; (iv) Sample loading bias was normalized with the trimmed median intensity of all PSMs; (v) the mean-centered intensities across samples was calculated, (vi) protein relative intensities by averaging related PSMs was calculated; (vii) protein absolute intensities by multiplying the relative intensities by the grand-mean of three most highly abundant PSMs was computed.

### Principal component analysis

Principal component analysis (PCA) was used to visualize the differences among samples. All gene and metabolite abundance were used as features of PCA. The pairwise Euclidean distance between features was calculated. PCA was performed using the R package prcomp (version 3.4.0).

### Differential expression analysis

Differentially expressed proteins between the two strains were identified using the limma R package (version 3.46.0). The Benjamini-Hochberg method for false discovery rate correction was used, and proteins with an adjusted *p*-value < 0.05 and log_2_ fold change > 1.5 were defined as differentially expressed between the B6 and D2 strains.

### Pathway enrichment

To assess the functional relevance of the differentially expressed proteins, the R package clusterProfiler (version 3.18.1) was used for gene ontology enrichment analysis. Gene ontology terms with a Benjamini-Hochberg adjusted *p*-value < 0.05 were defined as significantly enriched.

### Characterization of additive and dominance inheritance

The additive effect, A, is estimated as half of the observed difference between the parental strains. The dominance effect, D, was estimated as the difference between the F1 and the mid-parent values. We defined the scaled difference in expression levels between F1s and mid-parent strains as follows,

$$D/A=\frac{\left[\frac{\left(B6D2F1+D2B6F1\right)}{2}-\frac{\left(B6+D2\right)}{2}\right]}{\max \left(B6,D2\right)-\frac{\left(B6+D2\right)}{2}}$$

### Analysis of patterns of genetic inheritance

To estimate heritability, we first calculate the variance components for additive effect (A), dominant effect (D), sex effect (Vs), and residue (Ve). These variances were estimated using a mixed linear model with population dependence structures:

$$\mathrm{y}=\upmu +\mathrm{A}+\mathrm{D}+\mathrm{S}+\upvarepsilon$$

where y is a vector of *n* × 1 observations (i.e., the expression level of each protein); n is the number of samples across different mouse strains with different genders; μ is the model average; A is the random additive effect, which follows the distribution N $\left(0,{\upsigma}_{\mathrm{a}}^2{\mathrm{R}}_1\right)$, in which ${\upsigma}_{\mathrm{a}}^2$ is the additive genetic variance and R_1_ is the relatedness matrix of additive variance. D is the random dominant effect, which follows the distribution N $\left(0,\kern0.5em {\upsigma}_{\mathrm{d}}^2{\mathrm{R}}_2\right)$, in which ${\upsigma}_{\mathrm{d}}^2$ is the dominant genetic variance and R_2_ is the relatedness matrix of dominant variance. Relatedness matrix of genetic variance describes how individuals are genetically related to each other in terms of additive and dominant inheritance. These values are the expectations of the relatedness values derived from the pedigree structure. S is the fixed effect of sex, and ε is the residual error. Each variance component was estimated with Solving Mixed Model Equations in R (SOMMER) package.

R_1_ and R_2_ matrices are defined below:

$${\mathrm{R}}_1=\left[\begin{array}{cc}\begin{array}{cc}1& 0\\ {}0& 1\end{array}& \begin{array}{cc}0.5& 0.5\\ {}0.5& 0.5\end{array}\\ {}\begin{array}{cc}0.5& 0.5\\ {}0.5& 0.5\end{array}& \begin{array}{cc}1& 0.5\\ {}0.5& 1\end{array}\end{array}\right]$$

$${\mathrm{R}}_2=\left[\begin{array}{cc}\begin{array}{cc}1& 0\\ {}0& 1\end{array}& \begin{array}{cc}0& 0\\ {}0& 0\end{array}\\ {}\begin{array}{cc}0& 0\\ {}0& 0\end{array}& \begin{array}{cc}1& 0.25\\ {}0.25& 1\end{array}\end{array}\right]$$

Heritability was estimated as the proportion of total genetic variance for each protein:

$${H}^2=\frac{V_a+{V}_d}{V_a+{V}_d+{V}_s+{V}_e}$$

### Protein ASE detection

D2 SNPs (dbSNP version: 142) were downloaded from the UCSC genome browser database and were re-annotated using the genome annotation tool ANNOVAR [29] based on the GRCm38 (mm10) genome assembly. A customized protein database was constructed by appending mouse UniProt database with SNPs with the amino acid sequences of nonsynonymous variants. MS data were searched by JUMPg [19], a proteogenomic tool we recently developed. The false discovery rate (FDR) for variant peptide identification was set to 1% at the peptide level.

B6 variant peptides were identified from the original peptides quantified using JUMP if they contained a nonsynonymous variant. Variant peptides with their respective alleles detected in both B6 and D2 were retained for the detection of protein allele-specific expression (pASE). An empirical Bayes-moderated *t*-test between alleles with a Benjamini-Hochberg adjusted *p*-value < 0.05 was used to detect peptides displaying pASE. To compare *cis*- and *trans*- regulation of pASE, allelic expression ratios were calculated as log_2_(B6 peptide abundance) – log_2_(D2 peptide abundance) in both the parental strains and F1 strains.

### Transcriptomic analysis and ASE analysis at the transcript level

Paired-end RNA-seq data was downloaded from the European Nucleotide Archive for parental strains B6 and D2 whole brain tissue (accession number ERP000614) and for B6xD2 hybrid whole brain tissue (accession number ERP000591). Reads were trimmed to remove low-quality sequences using Trimmomatic (version 0.39), resulting in ~ 134 m read pairs for parental strains and ~ 148 m read pairs for the hybrid strain (2 × 30–76 bp).

A reference sequence for D2 was created using vcftools (version 0.1.17) by merging D2 SNPs with the current GRCm38 (mm10) reference assembly. Trimmed RNA-seq reads from all samples were aligned to both the consensus D2 and the GRCm38 reference sequences using STAR (version 2.7.1) with the parameter “--outFilterMultiMapNmax 1” to only retain uniquely mapped reads. Reads that aligned to regions containing SNPs were sorted based on mapping quality to either the B6 (GRCm38) or D2 allele using a python script [30]. Genes displaying ASE were identified in the hybrid samples using a binomial test with a Benjamini-Hochberg adjusted *p*-value < 0.05.

## Supplementary Information

**Additional file 1: Supplementary Table S1**: Proteins identified and quantified by LC/LC-MS/MS. **Supplementary Table S2:** Differentially expressed (DE) proteins in whole brain between B6 and D2 strains. **Supplementary Table S3:** Significantly enriched Gene Ontology (GO) terms of DE proteins between C57BL/6 J and DBA/2 J. **Supplementary Table S4:** Genetic variance and heritability estimates for all 9979 proteins identified. **Supplementary Table S5:** Variant peptides detected by the proteogenomics analysis. **Supplementary Table S6:** Peptides showing allele-specific expression.

**Additional file 2: Supplementary Figure S1**. Scatter plots showing correlation analysis of two replicates of mouse samples. R2 is the coefficient of determination. **Supplementary Figure S2**. Heat map showing differentially expressed proteins between four groups. **Supplementary Figure S3**. Enrichment analysis of proteins with higher expression in D2. **Supplementary Figure S4**. Comparison of protein expression between the two F1 hybrids (i.e. B6D2F1 and D2B6F1). **Supplementary Figure S5** Simulation analysis of statistical power to detect differentially expressed proteins in a proteome experiment.

## Data Availability

The datasets supporting the conclusions of this article are available in the ProteomeXchange database, with identifier PXD025830 and the European Nucleotide Archive with the accession numbers ERP000614 and ERP000591.

## Abbreviations

ASE: Allele-specific expression
B6: C57BL/6 J
CV: Coefficient of variation
CNV: Copy number variation
DEP: Differentially expressed protein
D2: DBA/2 J
FDR: False discovery rate
GO: Gene Ontology
LC/LC-MS/MS: Liquid chromatography coupled with tandem mass spectrometry
pASE: Protein allele-specific expression
PSM: Peptide spectrum match
QTLs: Quantitative trait loci
SILAC: Stable isotope labeling by amino acids in cell culture
SPE: Single parent expression
TMT: Tandem mass tag
