# Supplementary material for: Genetic architecture of protein expression and its regulation in the mouse brain
Source: BMC Genomics. 2021 Dec 4;22:875. doi: 10.1186/s12864-021-08168-y (PMC8642946; doi:10.1186/s12864-021-08168-y)
Supplement: Supplementary file 2 — Additional file 2: Supplementary Figure S1. Scatter plots showing correlation analysis of two replicates of mouse samples. R2 is the coefficient of determination. Supplementary Figure S2. Heat map showing differentially expressed proteins between four groups. Supplementary Figure S3. Enrichment analysis of proteins with higher expression in D2. Supplementary Figure S4. Comparison of protein expression between the two F1 hybrids (i.e. B6D2F1 and D2B6F1). Supplementary Figure S5 Simulation analysis of statistical power to detect differentially expressed proteins in a proteome experiment. [file 12864_2021_8168_MOESM2_ESM.pdf]

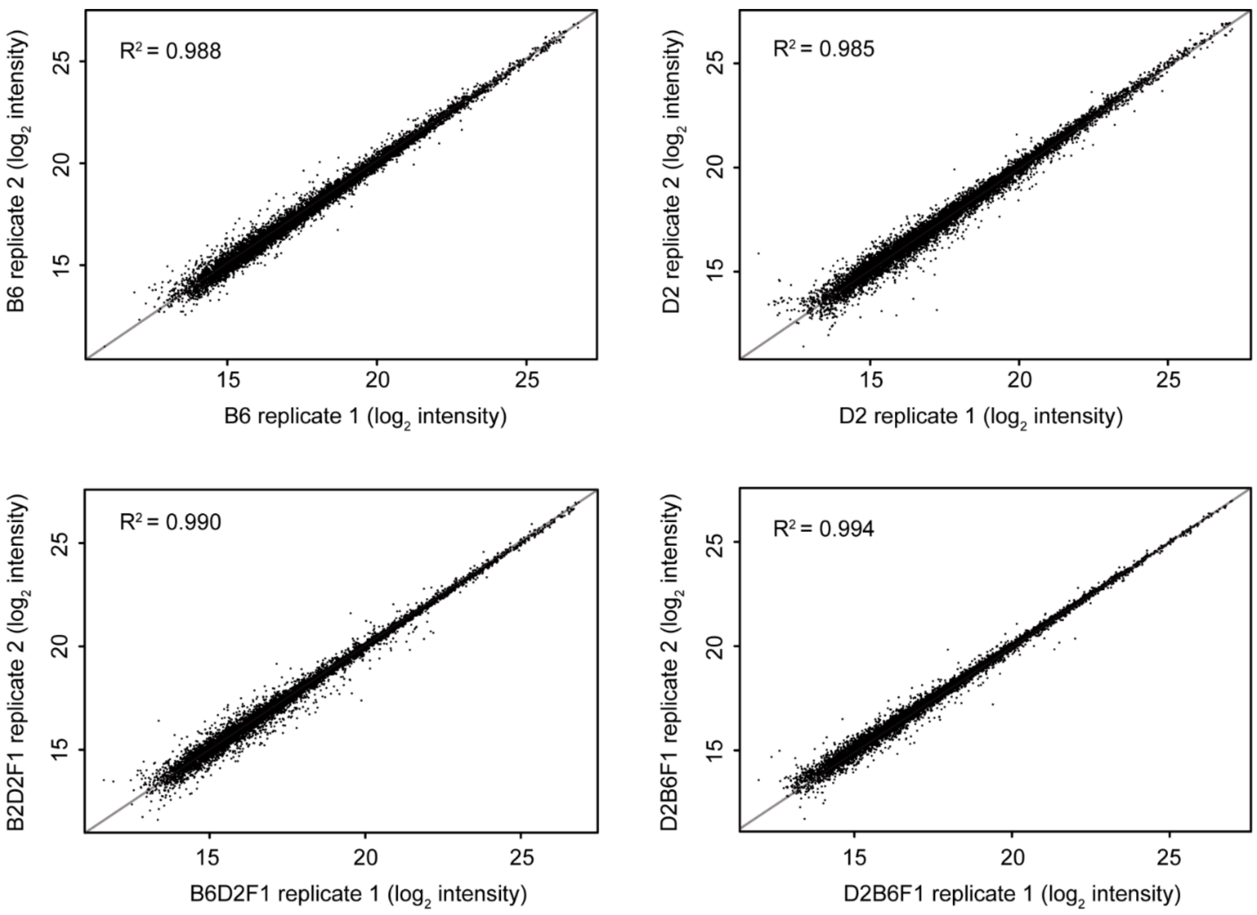

Supplementary Figure 1. Scatter plots showing correlation analysis of two replicates of mouse samples. R^2 is the coefficient of determination.

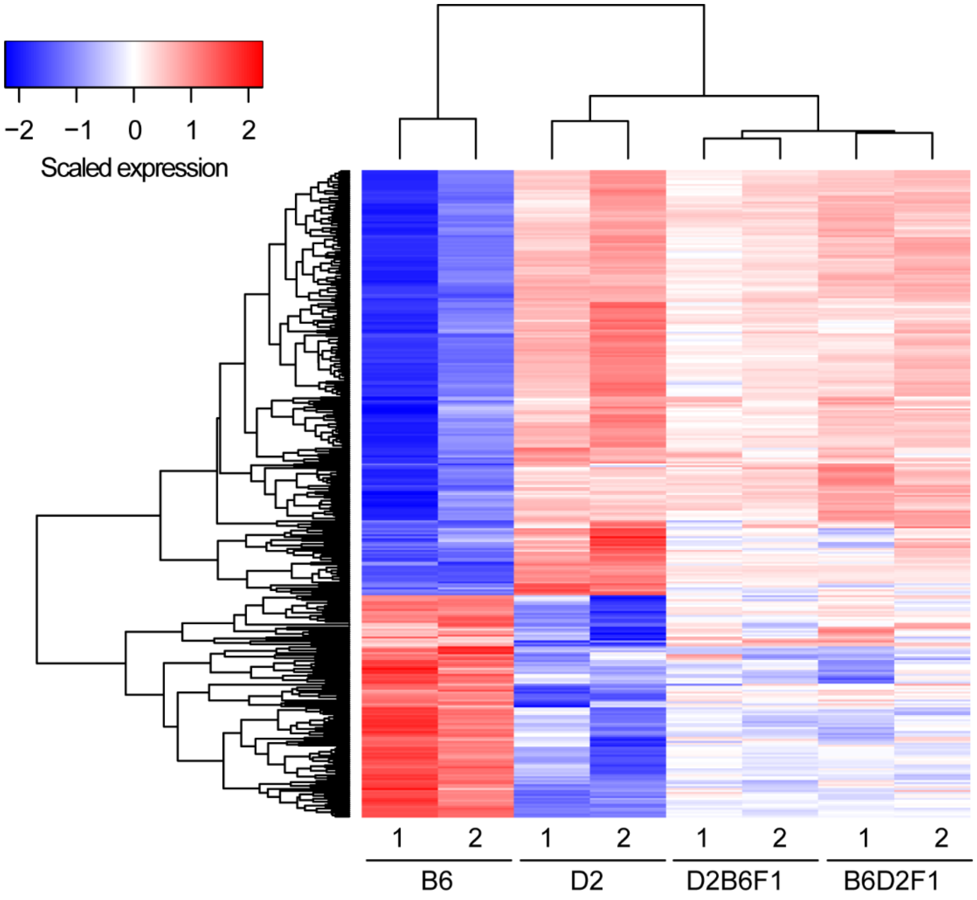

Supplementary Figure 2. Heatmap showing differentially expressed proteins between four groups.

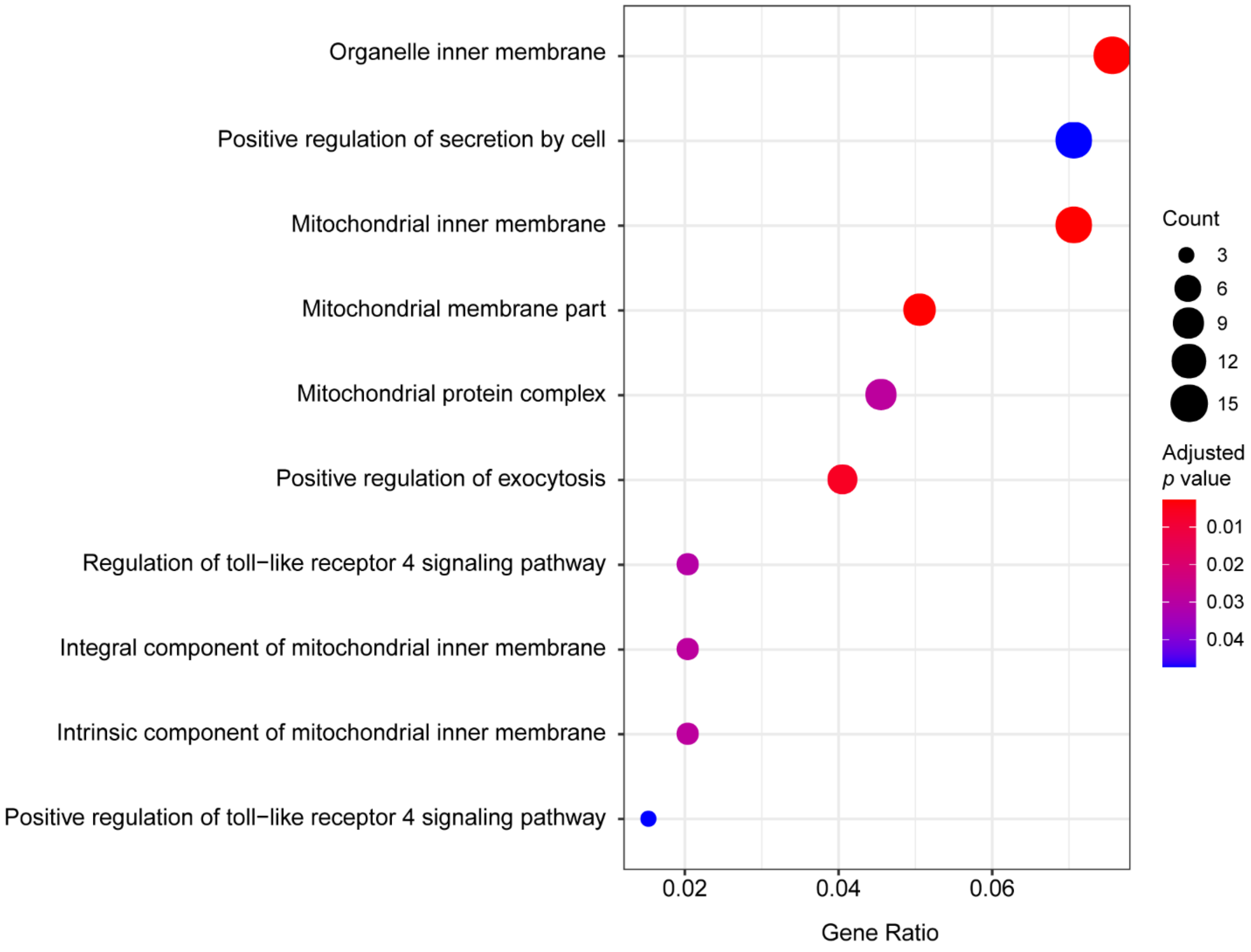

Supplementary Figure 3. Enrichment analysis of proteins with higher expression in D2.

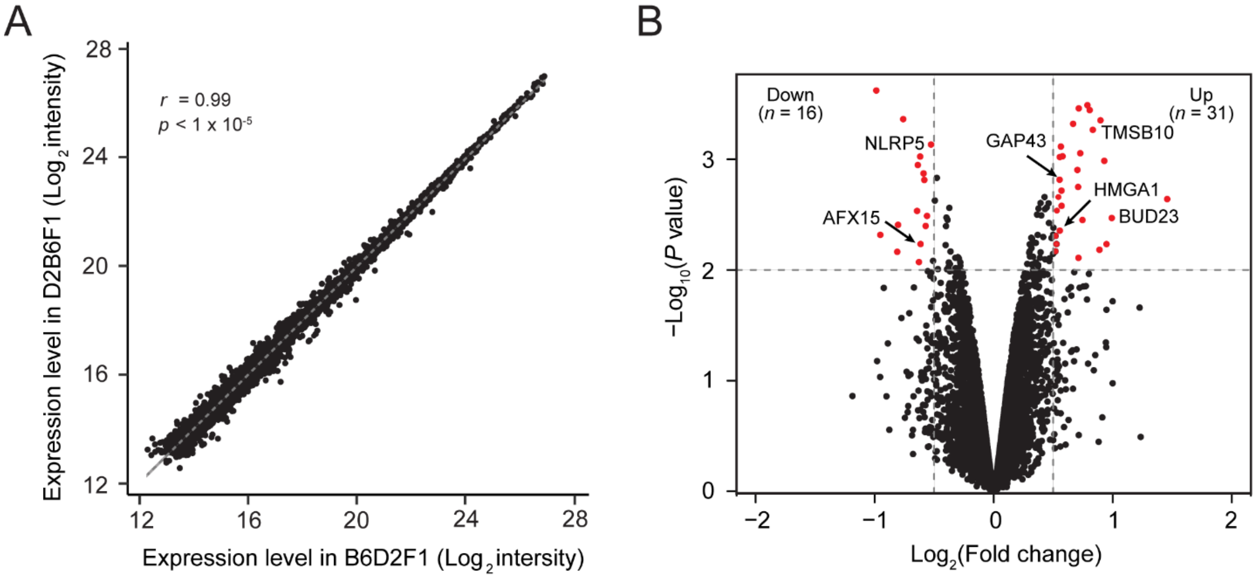

Supplementary Figure 4. Comparison of protein expression between the two F1 hybrids (i.e. B6D2F1 and D2B6F1). (A) Scatter plot showing a high correlation of expression between B6D2F1 and D2B6F1. (B) Volcano plot of protein expression differences between B6D2F1 and D2B6F1. Proteins highlighted in red are those with significant difference between the two F1 hybrids. Six proteins labeled with gene name are potentially associated with imprinting.

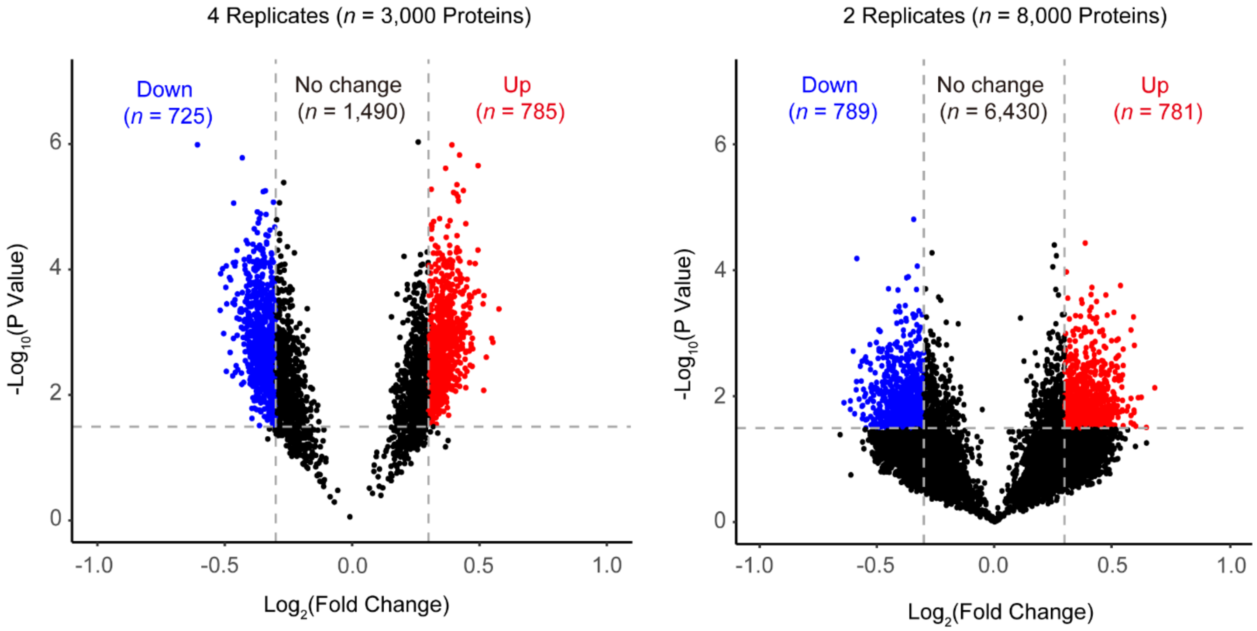

Supplementary Figure 5. Simulation analysis of statistical power to detect differentially expressed proteins in a proteome experiment. Scatter plot showing the number of differentially expressed proteins from a shallow proteome (3,000 proteins with 4 replicates) and a deep proteome (8,000 proteins with 2 replicates).
